# *In Vivo* Evaluation of Plane Wave Imaging for Abdominal Ultrasonography

**DOI:** 10.3390/s20195675

**Published:** 2020-10-05

**Authors:** Sua Bae, Jintae Jang, Moon Hyung Choi, Tai-Kyong Song

**Affiliations:** 1 Department of Electronic Engineering, Sogang University, Seoul 04107, Korea; suabae@sogang.ac.kr (S.B.); jterry123@sogang.ac.kr (J.J.); 2 Department of Radiology, College of Medicine, The Catholic University of Korea, Seoul 03312, Korea

**Keywords:** medical diagnostic imaging, ultrasonic imaging, abdominal ultrasound, plane wave imaging, diverging wave imaging, synthetic focusing

## Abstract

Although plane wave imaging (PWI) has been extensively employed for ultrafast ultrasound imaging, its potential for sectorial B-mode imaging with a convex array transducer has not yet been widely recognized. Recently, we reported an optimized PWI approach for sector scanning that exploits the dynamic transmit focusing capability. In this paper, we first report the clinical applicability of the optimized PWI for abdominal ultrasonography by in vivo image and video evaluations and compare it with conventional focusing (CF) and diverging wave imaging (DWI), which is another dynamic transmit focusing technique generally used for sectorial imaging. In vivo images and videos of the liver, kidney, and gallbladder were obtained from 30 healthy volunteers using PWI, DWI, and CF. Three radiologists assessed the phantom images, 156 in vivo images, and 66 in vivo videos. PWI showed significantly enhanced (*p* < 0.05) spatial resolution, contrast, and noise and artifact reduction, and a 4-fold higher acquisition rate compared to CF and provided similar performances compared to DWI. Because the computations required for PWI are considerably lower than that for DWI, PWI may represent a promising technique for sectorial imaging in abdominal ultrasonography that provides better image quality and eliminates the need for focal depth adjustment.

## 1. Introduction

Abdominal ultrasound (US) requires a large field-of-view with high image quality at all depths because abdominal organs examined by US imaging are of various sizes and located at various depths [1]. For example, the gallbladder and common bile duct, which are located at shallow depths (2–7 cm), need to be reconstructed with a sufficiently high spatial resolution to estimate the wall thickness, while the liver and kidney, which are located at both shallow and deep depths (5–20 cm), also require high spatial and contrast resolutions. When conventional focusing (CF) is used, however, the transmit beam has a fixed focal depth, which exhibits a higher spatial resolution and contrast of the image in the vicinity of the focal depth but lower image quality at other depths. Consequently, when scanning the entire abdomen, clinicians must constantly adjust the focal depth up and down to the region of interest with one hand while holding a transducer with the other hand. This constant manual adjustment of the focus prolongs the examination time.

A simple method of enhancing the image quality over various depths is to increase the number of transmit foci per scanline [2]. In the multifocus technique, multiple beams focused at different depths are successively transmitted to reconstruct a single scanline of an image. Consequently, the frame rate decreases inversely proportional to the number of foci. Although this technique is commonly used for linear array imaging with a short depth of view (<8 cm), it is difficult to apply for abdominal US imaging when the view depth is greater than 15 cm because of the long acquisition time. If three foci per scanline are used for a B-mode image with 256 scanlines and a depth of 20 cm, then the frame rate will be lowered to 5 Hz, which is extremely slow for real-time US scanning. 

Another method is synthetic transmit focusing (STF), in which multiple low-resolution images obtained by transmitting an unfocused beam or a widely diverging beam before and after a tight focus are compounded coherently to achieve dynamic transmit focusing at every imaging point. Synthetic aperture (SA) imaging is the most investigated and widespread STF technique. In SA imaging, a virtual source (VS) is usually generated in front of the array transducer, and spherical wavefronts before and after the VSs are used for STF [3,4,5]. Because it was adapted for medical imaging in the 1980s–90s [3,6,7], numerous studies have been published and verified that SA imaging provides a high-resolution image over all depths compared to conventional focusing [8,9,10,11]. Consequently, it has been implemented as an innovative advanced beamformer on commercial high-end US systems, such as *n*SIGHT by Philips or cSound by GE [12,13]. However, this technique requires reconstruction of dozens of hundreds of more scanlines per frame compared to the CF method, which substantially increases the computational costs. In addition, it suffers from motion artifacts because high-quality SA imaging often requires approximately 100 emissions due to grating lobe problems [8,14], and tissue or hand motion could cause incoherence among the low-resolution images that are subsequently compounded and lower the performance of dynamic transmit focusing. Moreover, motion artifacts can be more problematic in abdominal ultrasonography due to the large field-of-view and long acquisition time, which hinders the fast scanning and capturing of various abdominal organs.

When VSs are set behind the transducer array, diverging waves (DWs) are transmitted through multiple elements covering a broad imaging region. In DW imaging (DWI), a small number of DWs, usually 3–20, with different VS positions in the lateral direction are transmitted and coherently compounded for a single frame [15,16,17], thus leading to fast acquisition and less motion artifact. Using the broad coverage of DW, DWI is generally used for fast cardiac imaging with a phased array [16,18,19,20,21]. Because DWI offers dynamic transmit focusing with a small number of emissions, it can also be used to enhance the image quality of abdominal US, avoiding significant motion artifacts. Consequently, a recent paper suggested the use of DW for ultrafast abdominal imaging [22].

When the VSs are placed at infinity behind the array, plane waves (PWs) are transmitted. PW imaging (PWI) employs STF using the PWs steered at different angles [23]. It has been widely used as a means of ultrafast imaging for shear wave elastography [24,25,26] and blood flow imaging [27,28] or fast 3D scanning [29,30,31]. Although most PWI studies focus only on its ultrafast imaging capability, a few studies have reported its potential for high-resolution B-mode imaging [23,26,32]. In addition, PWI is more advantageous than DWI in terms of the spatial resolution [19,22] because PW STF can theoretically provide a uniform beam width over all depths [26,33]. However, PWI has not been widely used for large sectorial imaging because of the small coverage of PW compared to that of DW, which might be one of the reasons why PWI has not been implemented for abdominal imaging. To test the feasibility of PWI for this unexplored application, in our earlier study [34,35], we optimized PWI for the sectorial field-of-view and conducted simulation and phantom experiments. From that study, PWI was proven to enhance both the image quality and frame rate in convex array imaging, which results similar to that of linear array imaging when the PW angles, transmit aperture size, and synthesized PWs for each imaging point were properly selected.

The objective of this paper is to test the clinical applicability of the optimized PWI for abdominal ultrasonography by evaluating its in vivo image quality and comparing its quality with that of DWI and CF. To the best of our knowledge, this is the first in vivo study of PWI for abdominal ultrasonic imaging. Phantom images, in vivo images, and videos of the liver, kidney, and gallbladder of 30 healthy volunteers were obtained using PWI, DWI, and CF. First, the phantom images were used to measure the spatial resolution and image contrast for the quantitative evaluation. Then, in vivo abdominal US images were acquired from 30 healthy volunteers and assessed by three radiologists in terms of spatial resolution, image contrast, noise, and artifacts. In addition, in vivo video clips were also evaluated by radiologists to assess image quality under hand motion.

## 2. Materials and Methods

### 2.1. Imaging Techniques

Three imaging techniques were compared: CF, DWI, and PWI. The transmit configuration of each imaging technique is listed in Table 1. In CF imaging, traditional line-by-line scanning was performed using a focused beam with a focal depth of 10 cm and an F-number of 5.0. The focal depth and the F-number were optimized so that the spatial resolution was as constant with increasing depth as possible to obtain uniform spatial resolution in the entire field-of-view for the fair comparison with two-way dynamic focusing techniques (DWI and PWI). In addition, 128 focused beams were used for CF, and 32 VSs and 32 PWs were employed for DWI and PWI, respectively.

**Figure 1:**
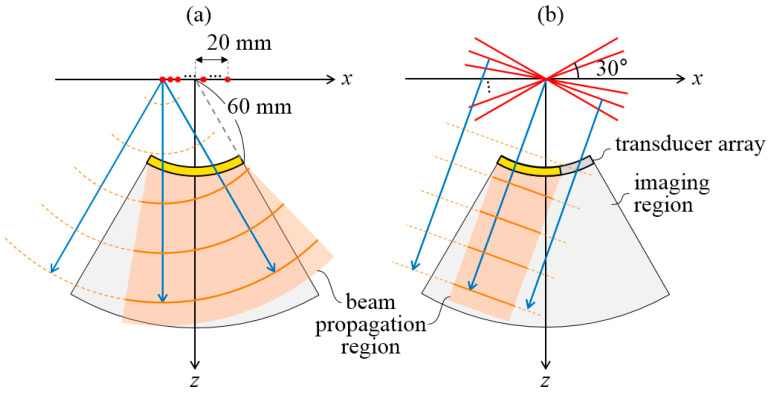
(**a**) Virtual sources (VSs) (red dots) and propagating DW (orange arcs) used for DWI and (**b**) steered PWs (solid red lines) and propagating PW (orange lines) used for PWI. Directions of DWs and PWs are indicated by blue arrows, and transmit apertures are marked with yellow shaded areas. Pixels in the beam propagation region (orange shaded area) for each DW or PW transmission should be reconstructed in the beamforming process. Difference in the size of beam propagation region between DWI and PWI shows the difference in the amount of computations required for DWI and PWI.

Figure 1 illustrates the VSs used for DWI and PWs used for PWI. The red dots in Figure 1a show the 32 VSs, and the orange arcs represent the propagation of a DW originated from the first VS over time. The red lines in Figure 1b show the 32 steered PWs, and the orange lines illustrate the propagating PW. The blue arrows in Figure 1 show the directions of the DW and PW. The VS positions (Table 1) were chosen to employ the full aperture (yellow shaded area in Figure 1a) for each DW transmission. The outermost VS was positioned at *x* = ± 20 mm (Figure S1) considering the 6 dB acceptance angle of the element of the convex array transducer used in this experiment. In PWI, the transmit aperture size was limited, indicated by a yellow shaded area in Figure 1b, such that it did not exceed the acceptance angle of the transducer element for the optimal PWI as proposed in our previous study [35].

In sector imaging, PW has a smaller beam propagation region (smaller coverage) compared with DW (orange shaded areas in Figure 1) because of the limited transmit aperture and a constant direction of propagation. Although this property of PW reduces the number of synthesized waves per imaging pixel, it does not diverge and maintains the wave intensity throughout the propagation, leading to a deeper penetration depth compared with that of DW. More importantly, the small propagation region reduces the amount of computations required in beamforming. Table 1 shows the normalized amount of computations for each imaging technique when a B-mode image with 1039 × 256 pixels was reconstructed. This value was obtained by normalizing the number of beamforming (channel-summation) operations required for a single compounded image using DWI or PWI by the value required for a single image using CF. As is well known, synthetic imaging (DWI and PWI) requires much more computations than traditional focusing (CF). Note that PWI with 32 PWs requires 2.9-times fewer computations compared with DWI with 32 VSs as shown in Table 1.

Given the imaging depth *d*, the sound speed *c*, and the time margin before the next emission *τ*, the acquisition rate for a single frame can be calculated by

(1) $${\mathrm{FR}}_{\max}\;=\;1/\left\{\left(2d/c+\tau \right)\cdot N_{\mathrm{tx}}\right\}$$

The acquisition frame rates of CF, DWI, and PWI are 31.9 fps, 127.7 fps, and 127.7 fps, respectively, when *d* = 15 cm, *c* = 1540 m/s, and *τ* = 50 *μs* (Table 1). However, during the in vivo data acquisition with the US system, the display frame rates (i.e., the frame rate at which the B-mode image was updated on the screen) were 26.6 fps, 10 fps, and 15 fps for CF, DWI, and PWI, respectively, which were lower than the acquisition rates due to the limited number of receive channels and computing power of the system. Although 2.9 times fewer computations were required for PWI than DWI (Table 1), the display frame rate of PWI supported by the system was only 1.5 higher than that of DWI due to the limited channel count of the system. The reasons for the low display frame rate will be further explained in discussion section. Note that the display frame rate could, however, be enhanced up to the acquisition frame rate by improving the computational algorithms in the beamforming process and upgrading the computational resources of the US system.

For all the imaging techniques, the transmit voltage was 80 V, the receive F-number was 1.0, and the 50% Tukey window was used for the receive apodization in the beamforming process. In DWI and PWI, only the imaging points reached by the DW or PW were calculated as the low-resolution image and compounded for the final image as described in [35].

### 2.2. System and Method for Data Acquisition

A research US system (E-cube 12R, Alpinion Medical Systems, Republic of Korea) with a convex array transducer (SC1-6, Alpinion Medical Systems, Republic of Korea) was used for data acquisition. The transducer has 128 elements and a center frequency of 3.6 MHz, and the system has a 128-channel transmit board and a 64-channel receive board. For DWI and PWI, which require full-channel reception, the same emission was repeated twice for the echo reception of the first and second sets of 64 channels. Beamforming and image processing were conducted on a graphics processing unit (GPU) (GeForce GTX 1080, NVIDIA, CA, USA) equipped in the system by using the CUDA computing platform. Thus, unfortunately, the acquisition frame rate of DWI and PWI in this study was twice the maximum acquisition frame rate (127.7/2 = 63.85 fps).

A commercial phantom (Model 539, ATS laboratories Inc., Bridgeport, CA, USA) was used for the phantom study. For the phantom images of CF, DWI, and PWI, radio-frequency (RF) data were acquired by fixing the transducer on the phantom. A cross-section of the phantom including point and cyst targets was selected, and three images of the same cross-section were reconstructed using the three imaging techniques (CF, DWI, and PWI).

In vivo abdominal ultrasonic images of the gallbladder, liver, and kidney were collected from 30 healthy male volunteers by a radiologist under institutional review board approval at Seoul Saint Mary’s Hospital. Written informed consent was obtained from all volunteers. The radiologist obtained abdominal ultrasonic images and videos of each volunteer using CF, DWI, and PWI, sequentially, trying to obtain three images or videos (CF, DWI, and PWI) for the same cross-section as much as possible. Misaligned sets were excluded, and 52 image sets (a total of 156 images) were evaluated: 14 sets for the gallbladder, 18 sets for the liver, and 20 sets for the kidney. For the video evaluation, 22 video sets (a total of 66 clips) were chosen, and each video contains the real-time image of right hepatic lobe, gallbladder, and right kidney. Three video clips (CF, DWI, and PWI) of each set were synchronized to show the same cross-section at the same time point as much as possible. The time length of the synchronized videos was between 3 and 9 s.

### 2.3. Beamforming and Postprocessing of Image for the Evaluation

For the still images, the RF channel data were stored and beamforming and postprocessing were conducted offline. In the beamforming process, the RF data were demodulated to the base band, downsampled by a factor of 4, and then beamformed using the parameters shown in Section 2.1. To flatten the uneven brightness of the image across depths within an image and across different imaging techniques, automatic time-gain-compensation (TGC) was applied to all the images as in [8]. The imaging region was axially divided into 5 zones, and the 5 representative gain values were determined by the reciprocal of the median brightness of each zone. TGC was applied after the spline interpolation of the 5 gain values.

In the log compression, which highly affects the contrast of an image, the max value was automatically chosen to be 50 dB and 40 dB above the median brightness of the entire image for the phantom and in vivo images, respectively. The dynamic range was 80 dB and 57 dB for the phantom and in vivo images, respectively.

The RF channel data for in vivo videos could not be stored due to the limited storage capacity. The videos were obtained by recording displayed B-mode images on the screen. Because the automatic TGC was not implemented on the online reconstruction software in the system and the radiologist arbitrarily adjusted the gain during the acquisition, the brightness of the on-screen images among DWI, PWI, and CF was quite different. Thus, the automatic TGC was applied on a log scale to the recorded video clips. For this reason, unfortunately, the image contrast of video could not be evaluated because the brightness of the screen-captured video was already clipped with different ranges before the post TGC control. 

### 2.4. Image and Video Evaluation

Three radiologists with 10 years, 8 years, and 5 years of abdominal ultrasonography experience assessed the phantom images and the in vivo images and videos of the human abdomen. The radiologists were asked to score each image or video on a 5-point Likert scale (1: very poor, 2: poor, 3: average, 4: good, and 5: very good) in terms of 4 evaluation items (‘spatial resolution’, ‘contrast’, ‘noise’, and ‘artifacts’). The videos were not assessed in terms of ‘contrast’ because some grayscale values were saturated due to the unavailability of raw data as described in Section 2.3.

In the phantom study, 3 images (1 set) of a cross-section of the phantom were reconstructed using CF, DWI, and PWI. The 3 images were randomly ordered without labels and presented to evaluators. For the in vivo study, 156 images (52 sets) were randomly ordered and evaluated individually without any information about the patients and imaging techniques. For the assessment of in vivo videos (22 sets), the three synchronized videos of each set were played together side by side with random order. The radiologist could rewind and play back the videos freely during the assessment.

For the phantom study, the spatial resolution and contrast were also quantitatively measured. The spatial resolution was measured by the lateral length of the –6 dB contour of a point target [35]. The contrast ratio was calculated by *CR* = $\mu_{b}-\mu_{c}$, where $\mu_{b}$ and $\mu_{c}$ are the mean intensities of the background speckle and cyst regions, respectively [36].

### 2.5. Statistical Analysis

The Wilcoxon rank-sum test was used because it is known to be suitable for a Likert scale evaluation [37,38]. Because the absolute Likert scale values highly depend on the person’s interpretation of the scale, the test was applied to each evaluator’s scores. Three pairs of data (CF versus (vs.) DWI, CF vs. PWI, and DWI vs. PWI) were tested to statistically demonstrate that PWI offers a better image quality than does CF imaging and provides comparable performance to DWI. The mean score difference between two among three imaging techniques were obtained. For example, the mean score difference between PWI and DWI (P vs. D) was calculated as

(2) $$d_{P\;\mathrm{vs}.\;D}\;=\;\frac{1}{N}\sum_{n}s_{P}\left(n\right)-s_{D}\left(n\right)$$

where $s_{P}\left(n\right)$ and $s_{D}\left(n\right)$ are scores of *n*-th image or video clip reconstructed by PWI and DWI, respectively.

## 3. Results

### 3.1. Phantom Study

**Figure 2:**
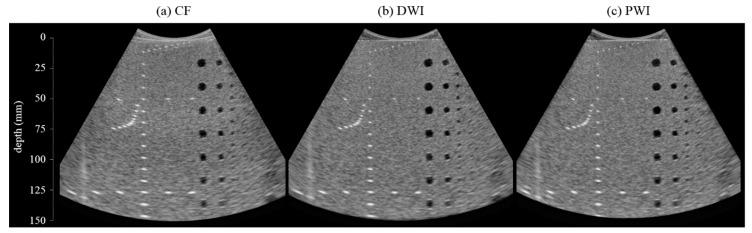
Phantom image set reconstructed by using (**a**) CF, (**b**) DWI, and (**c**) PWI. The enlarged images of point and cyst targets are shown in Figure 3 and Figure 4.

**Figure 3:**
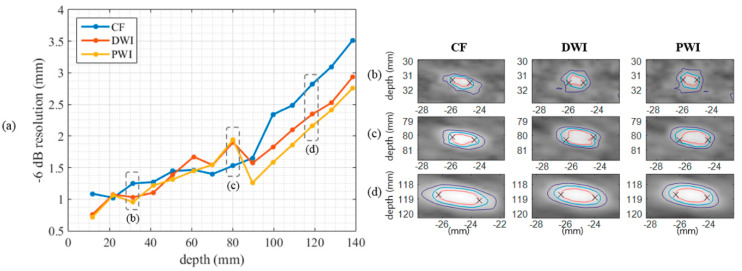
(**a**) Resolution (mm) over depths measured from point targets in the phantom images of CF, DWI, and PWI. DWI and PWI provided a better resolution at depths ≥ 100 mm. (**b**–**d**) Magnified point target images of Figure 2 at depths of (**b**) 30 mm, (**c**) 80 mm, and (**d**) 120 mm. Red, blue, and purple lines show −6 dB, −12 dB, and −20 dB contours, respectively, and the two crosses in each panel indicate where the maximum lateral distance is measured as the resolution value.

Figure 2 shows the phantom images reconstructed by the CF, DWI, and PWI techniques. The –6 dB spatial resolutions of the point targets in Figure 2 are presented in Figure 3a, and magnified images of the point targets at 30, 80, and 120 mm are presented with −6 dB, −12 dB, and −20 dB contours in Figure 3b–d. In Figure 3a, the effective focal depth of CF seems slightly closer to the transducer than 100 mm because the point targets were vertically located 2.5 mm apart from the center scanline. For all imaging techniques, the spatial resolution deteriorates as the depth increases. Although the resolutions of the three techniques are similar at shallow depths, PWI and DWI clearly show a better spatial resolution than that of CF at depths ≥ 100 mm. In addition, PWI provides a slightly better resolution than that of DWI. This result might be explained by the nondiffraction property of PWs [26,33], and the superiority of PWI over DWI in terms of spatial resolution was also previously reported [19,22].

**Figure 4:**
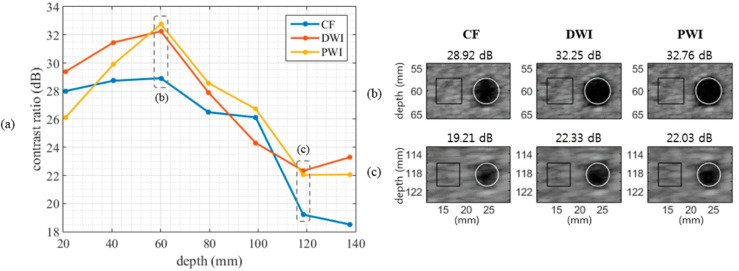
(**a**) Contrast ratio (dB) over all depths measured from cyst targets in the phantom images of CF, DWI, and PWI. The contrast ratio of CF is lower than those of DWI and PWI on average. (**b**, **c**) Magnified cyst target images of Figure 2 at depths of (**b**) 60 mm and (**c**) 120 mm. The black square and the white circle show the contrast measurement areas. The measured contrast ratio is presented above each panel.

The measured contrast of the cyst targets in Figure 2 is presented in Figure 4a, and the magnified cyst images are shown in Figure 4b,c. Except at a depth of 20 mm, PWI always offered a higher contrast ratio than CF. Compared with DWI, PWI provided a lower contrast at near depths but a similar contrast at mid and far depths. At near depths, DW had sufficient intensity before diverging further and the number of compounded DWs is greater than the number of compounded PWs (due to the broader coverage of a DW than a PW). This might lead to the higher contrast ratio of DWI than that of PWI at near depths (*z* = 20 and 40 mm). However, DWs lose the intensity more than PWs as it propagated. At mid and far depths (*z* = 60–140 mm), the contrast ratio of DWI and PWI became similar, although the number of compounded DWs is still greater than the number of compounded PWs. Both DWI and PWI showed the contrast degradation at near depths relative to the far depths. This might be due to the reverberation artifacts that appear more frequently in imaging techniques using broad beams than in conventional imaging. The mean contrast ratios of CF, DWI, and PWI were 25.15 dB, 27.27 dB, and 26.88 dB, respectively.

Table 2 lists the scores of phantom images of one set (Figure 2) reconstructed using CF, DWI, and PWI, and they were evaluated by three radiologists with consideration of the four items. PWI was scored higher than CF in all cases. All radiologists gave almost the same scores to DWI and PWI. From this phantom study, PWI was proven to provide better image quality with a higher acquisition rate than CF and to yield a comparable performance to DWI with much lower computational costs.

### 3.2. In Vivo Study

**Figure 5:**
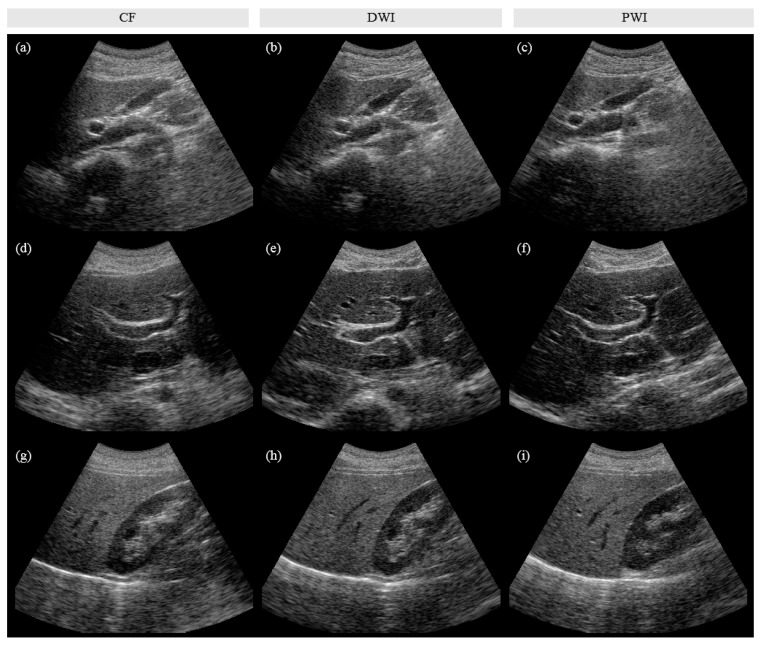
Representative in vivo images of (**a**–**c**) gallbladder, (**d**–**f**) liver, and (**g**–**i**) kidney, which were reconstructed by using CF (for (**a**, **d**, **g**)), DWI (for (**b**, **e**, **h**)), and PWI (for (**c**, **f**, **i**)). PWI showed better image quality with a 4-fold higher acquisition frame rate than CF and provided a comparable performance with a 2.9 times lower number of computations compared to DWI.

**Figure 6:**
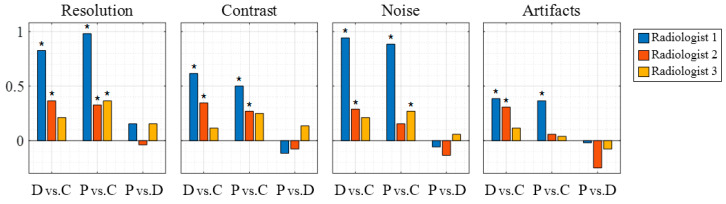
Mean score differences of in vivo images (*N* = 52) between DWI and CF (${\bar{d}}_{D\;\mathrm{vs}.\;C}$), PWI and CF (${\bar{d}}_{P\;\mathrm{vs}.\;C}$), and PWI and DWI (${\bar{d}}_{P\;\mathrm{vs}.\;D}$). The asterisk indicates a significant difference (*p* < 0.05). DWI and PWI show higher scores than CF in all cases, with significant differences in some cases. PWI and DWI show a similar performance with no significant differences.

Figure 5 shows representative gallbladder, liver, and kidney images obtained using CF, DWI, and PWI. Although slightly different cross-sections were captured across the imaging techniques, the overall image quality, including the spatial resolution and image contrast, is better in DWI and PWI than in CF. Figure 6 shows the clinical evaluation results of 156 in vivo images (52 sets) by the three radiologists. A bar represents the mean of differences in scores between DWI and CF ($d_{D\;\mathrm{vs}.\;C}$), PWI and CF ($d_{P\;\mathrm{vs}.\;C}$), and PWI and DWI ($d_{P\;\mathrm{vs}.\;D}$) obtained by (2). The *p*-values of the statistical test are listed in Table 3, and the significant differences (*p* < 0.05) are shown in bold in Table 3 and marked by an asterisk in Figure 6. Both DWI and PWI show higher scores than CF for all evaluation items by all three radiologists. DWI and PWI received very similar scores. Although PWI shows slightly better spatial resolution and DWI presents slightly higher scores for other image qualities, significant differences are not observed. 

Radiologist 1 found a highly significant enhancement (*p* < 0.01) of the ‘spatial resolution’, ‘contrast’, and ‘noise’ and a significant improvement (*p* < 0.05) in the ‘unwanted artifacts’ for the images obtained via DWI and PWI compared with those obtained via CF. Radiologist 2 also noted a significant enhancement (*p* < 0.05) of the ‘resolution’ and ‘contrast’ for DWI and PWI. Radiologist 3 indicated that PWI provides significantly better image quality (*p* < 0.05) based on the ‘resolution’ and ‘noise’ than CF. None of the radiologists found significant differences (*p* > 0.1) between DWI and PWI with respect to all the evaluation items.

**Figure 7:**
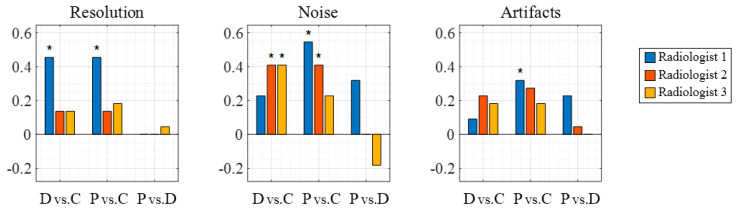
Mean of the score differences of in vivo videos (*N* = 22) between DWI and CF (${\bar{d}}_{D\;\mathrm{vs}.\;C}$), between PWI and CF (${\bar{d}}_{P\;\mathrm{vs}.\;C}$), and between PWI and DWI (${\bar{d}}_{P\;\mathrm{vs}.\;D}$). The asterisk indicates a significant difference (*p* < 0.05). DWI and PWI show higher scores on average than those of CF in all cases. PWI and DWI (P vs. D) show similar performance with no significant differences.

Figure 7 shows the clinical evaluation results of the in vivo videos by the three radiologists. The representative video is available online as multimedia Video S1. The *p*-values of the rank-sum test of scores are listed in Table 4, and the significant differences (*p* < 0.05) are shown in bold in Table 4 and marked by an asterisk in Figure 7. As observed in the result of the still image assessment, PWI had higher average scores than CF in all cases and similar scores to that of DWI in the video evaluation. Radiologist 1 found that PWI significantly enhanced the image quality for all evaluation items (*p* < 0.05) compared to CF. Because noise is relatively easier to recognize from videos than from still images, two of the three radiologists indicated that the DWI and PWI videos showed a significant enhancement with respect to ‘noise’ compared with the CF videos. Significant differences were not observed between PWI and DWI.

## 4. Discussion

In this paper, we demonstrated that PWI 1) provides significantly enhanced image quality with a 4-fold higher acquisition rate compared to line-by-line CF and 2) provides a comparable performance with a 2.9 times lower number of computations compared to DWI, based on quantitative and qualitative evaluations of phantom and in vivo images. In the phantom study, the spatial resolution at depths ≥ 100 mm was enhanced (~0.5 mm) and the contrast of cyst targets was improved (~2 dB higher on average) when using DWI and PWI compared with CF (Figure 3 and Figure 4, Table 2). In the in vivo study, the radiologists assessed the still images of 52 sets and the video clips of 22 sets, including liver, gallbladder, and kidney. 

Comparing PWI and CF, in the image evaluation (Figure 5 and Figure 6), radiologist 1 rated PWI significantly higher than CF for all evaluation items and radiologists 2 and 3 recognized the significantly improved image quality of PWI in terms of ‘resolution’, ‘contrast’, and ‘noise’ items (*p* < 0.05). In the video evaluation (Video S1 and Figure 7), radiologist 1 found a significant enhancement in PWI in terms of ‘resolution’, ‘contrast’, and ‘noise’, while radiologist 2 found significant enhancements in terms of ‘noise’ compared to CF. 

In addition to enhanced image quality, the fast acquisition rate is another advantage of PWI compared to CF. As the numbers of transmissions of PWI are 4-times lower than that of CF (Table 1), the acquisition rates under the physical speed of US in tissues are 4-fold higher than that of CF. This advantage of using a small number of emissions reduces the likelihood of motion artifacts, such as blurring and distortion, which are major issues in synthetic imaging.

A comparison between PWI and DWI showed that PWI had slightly better spatial resolution and DWI had slightly better contrast and reduced noise and artifacts (Figure 6 and Figure 7). Similar results were reported by Tong et al. [19] and Kang et al. [22]. However, the score differences between PWI and DWI were quite small and none were significant (*p* > 0.1, Table 3 and Table 4). Therefore, these findings imply that PWI is able to provide a comparable image quality to DWI in sector imaging. 

More importantly, PWI required an approximately 3-times lower amount of computations (Table 1) relative to DWI. For sector imaging, DW is usually chosen to achieve dynamic transmit focusing, which might be related to the larger field-of-view of sector imaging compared with linear-scan imaging and the broader coverage region (beam propagation region in Figure 1) of DW compared with PW. In this paper, however, we found that PWI can provide comparable image quality with a much lower amount of computations compared to that of DWI when the PW angles and transmit aperture size are carefully selected as in [35].

### 4.1. Dependence on Evaluators

From the statistical analysis of the in vivo images and videos (Table 3 and Table 4), significant differences were found most often in the assessment of radiologist 1, while the least significant differences among the three radiologists were found for the evaluation results of radiologist 3. This outcome might be associated with the evaluators’ clinical experience. Radiologists 1, 2, and 3 had 10 years, 8 years, and 5 years of experience, respectively, and the most experienced radiologist gave the scores with the largest variance (variance in the image evaluation scores was 1.16, 0.79, and 0.79 for radiologists 1, 2, and 3, respectively). The more experienced radiologists might have assessed the images with greater confidence, resulting in more significant differences in many items.

### 4.2. Real-time Realization

Similar to other STF imaging techniques, DWI and PWI require massive computations because dozens of scanlines should be reconstructed per single transmission and reception event, while CF requires a one- or two-scanline reconstruction per event (Table 1). Thus, this computational load makes the real-time implementation of STF imaging challenging, although both DWI and PWI have a high acquisition frame rate. In this case, the lower number of computations of PWI compared with DWI can be beneficial. 

Parallel processors can be successfully utilized for STF imaging to accelerate the reconstruction process because beamforming intrinsically performs the same operation on multiple data points. Software-based beamformers based on GPUs have been widely employed for STF imaging [13,39,40] as well as for conventional B-mode imaging, functional imaging, or three-dimensional imaging [29,30,41,42]. We also utilized a GPU for fast reconstruction of DWI and PWI. Although the display frame rate (real-time frame rate) of the system used in this study fell short of the acquisition frame rate, the process could be accelerated if the system supports a full channel reception and the online B-mode reconstruction software is further optimized, such as by using concurrent data copy and kernel execution. Indeed, using GeForce GTX 1080, it took 41.3 ms and 14.6 ms to compute a single synthesized (i.e., compounded) frame from channel data for DWI with 32 VSs and PWI with 32 PWs, respectively. Considering that parallel computing and data transfer technology is rapidly advancing, PWI with at least a 60-fps frame rate will soon be achievable.

### 4.3. Limitation of this Study

Despite the fast acquisition rates of PWI and DWI (Table 1), the display frame rates of PWI and DWI were lower than that of CF (26.6 fps, 10 fps, and 15 fps for CF, DWI, and PWI, respectively) in this study due to the lack of channel count and computing power of the system. The low display frame rates of PWI and DWI were mainly because they (1) need the full-aperture reception (128 channels) and (2) require 10–30 times more computations (Table 1) than CF. In CF, 64 channels were sufficient to receive echoes of a focused US beam from a straight scan line. However, DWI and PWI required a full 128-channel reception to collect echoes of a wide US beam reflected from a broad region. Unfortunately, the system supports only 64 reception channels and thus two times more transmit-receive sequences were performed to obtain 128-channel data with the 64-channel system. In addition, despite the use of a GPU for beamforming, the data transfer time and image reconstruction time for PWI and DWI was longer than the US echo acquisition time, which further decreases the display frame rates of PWI and DWI. 

For the still image evaluation, the B-mode image was reconstructed offline from RF channel data stored and thus the frame rate of image was only affected by the limited number of receive channels. Hence, each still image of CF, PWI, and DWI was acquired at the rate of 31.9 fps, 63.85 fps, and 63.85 fps, respectively. For the video evaluation, the screen-captured videos were used, and thus the frame rate of video was the same as the display frame rate (26.6 fps, 10 fps, and 15 fps for CF, DWI, and PWI, respectively). Those limited frame rates of images and videos might have affected the evaluation results. Note that despite this unfavorable condition (lower frame rate than possible), DWI and PWI received better scores than CF. If the 128-channel acquisition is available, the motion artifacts in DWI and PWI would be further reduced. In addition, if the real-time reconstruction is realized and the reconstruction frame rate is close to the acquisition frame rate, the system noise presented in the B-mode image would also be reduced by frame averaging because more frames could be averaged within a fixed averaging time period the for image persistence.

Although we optimized parameters for each imaging (the focal depth and F-number of CF for a uniform resolution over depths, the VS positions of DWI for full-aperture transmission, and the PW angles and aperture size of PWI according to our previous study [35]), only a single set of parameters for each imaging technique was used to evaluate the image quality in this study. More exhaustive comparisons with changes in various parameters might be needed because the number and directions (or angles) of synthesized waves are major determinants of image quality in PWI and DWI.

## 5. Conclusions

We evaluated PWI against line-by-line CF imaging and another dynamic transmit focusing technique, DWI, through phantom and in vivo experiments. The phantom images and in vivo images and videos of the liver, kidney, and gallbladder of 30 healthy volunteers were assessed by three radiologists. PWI showed a significant enhancement (*p* < 0.05) of the spatial resolution, contrast, and noise and artifact reduction and presented a 4-fold higher acquisition rate compared to CF. PWI and DWI showed similar performance in in vivo images and video evaluations, although PWI showed a slightly better spatial resolution and DWI presented a slightly higher scores for other image qualities; however, significant differences were not found. With comparable performance to DWI, PWI can considerably lower the number of computations (approximately by 3 times in this study), which is the most challenging aspect for the realization of synthetic imaging. Therefore, we concluded that PWI represents a promising tool for abdominal ultrasonography by enhancing the spatial resolution and contrast from shallow to deep depths and realizing a higher acquisition rate.

## Figures and Tables

**Table 1:** Number of transmissions (*N*_tx_), acquisition frame rate considering the *N*_tx_, normalized amount of computations in the beamforming process, and Tx beam properties of conventional focusing (CF), diverging wave imaging (DWI), and plane wave imaging (PWI).

	*N*_tx_	Acquisition Frame Rate (fps)	Amount of Computation (Normalized)	Tx Beam Properties
**CF**	128	31.9	1	Focal depth: 10 cm, F-number: 5.0
**DWI**	32	127.7	29.26	VS positions: *x*: from −2 cm to 2 cm with an interval of 4/31 cm, *z*: same as the vertex of the convex array
**PWI**	32	127.7	9.94	PW angles from −30° to 30° with an interval of 60/31°

**Table 2:** Evaluation results of phantom images (Figure 2) reconstructed by CF, DWI, and PWI on the Likert Scale (1–5) from three radiologists (Rad.). DWI and PWI were scored higher than CF in most cases.

Items		Spatial Resolution			Contrast			Noise			Artifacts		
Imaging		CF	DWI	PWI	CF	DWI	PWI	CF	DWI	PWI	CF	DWI	PWI
Score	Rad. 1	2	5	4	3	5	5	2	4	5	3	5	5
	Rad. 2	2	5	4	3	4	4	2	4	4	3	4	4
	Rad. 3	3	4	4	3	4	4	3	4	4	3	4	4

**Table 3:** P-values of the rank-sum test between each pair of CF, DWI, and PWI in the in vivo image evaluation (*N* = 52) (all P-values less than 0.05 (i.e., statistically significant differences) are shown in bold).

	Spatial Resolution			Contrast			Noise		
	D vs. C	P vs. C	P vs. D	D vs. C	P vs. C	P vs. D	D vs. C	P vs. C	P vs. D
Rad. 1	**0.000**	**0.000**	0.273	**0.002**	**0.012**	0.744	**0.000**	**0.000**	0.622
Rad. 2	**0.011**	**0.039**	0.691	**0.005**	**0.045**	0.829	**0.010**	0.111	0.887
Rad. 3	0.142	**0.024**	0.155	0.237	0.053	0.193	0.070	**0.041**	0.354
	**Artifact**								
	**D vs. C**	**P vs. C**	**P vs. D**						
Rad. 1	**0.020**	**0.029**	0.499						
Rad. 2	**0.027**	0.396	0.954						
Rad. 3	0.194	0.350	0.666						

Rad. = radiologist; D = DWI; P = PWI; C = CF.

**Table 4:** P-values of the rank-sum test between each pair of CF, DWI, and PWI in the in vivo video evaluation (*N* = 22) (all P-values less than 0.05 (i.e., statistically significant differences) are shown in bold).

	Spatial Resolution			Noise			Artifact		
	D vs. C	P vs. C	P vs. D	D vs. C	P vs. C	P vs. D	D vs. C	P vs. C	P vs. D
Rad. 1	**0.025**	**0.035**	0.531	0.121	**0.013**	0.093	0.224	**0.021**	0.102
Rad. 2	0.259	0.283	0.552	**0.016**	**0.021**	0.479	0.136	0.092	0.408
Rad. 3	0.261	0.155	0.359	**0.041**	0.106	0.807	0.146	0.146	0.506

## Supplementary Materials

The following are available online at https://www.mdpi.com/1424-8220/20/19/5675/s1, Figure S1: We placed the outermost VSs ± 20 mm from the center so that the full aperture can be used for the DW transmission within the acceptance angle of the transducer element. Video S1: Representative in vivo abdominal US videos reconstructed by CF, DWI, and PWI (from left to right).
