# Supplementary figures and images for: In Vivo Evaluation of Plane Wave Imaging for Abdominal Ultrasonography
Source: Sensors (Basel). 2020 Oct 5;20(19):5675. doi: 10.3390/s20195675 (PMC7584017; doi:10.3390/s20195675)

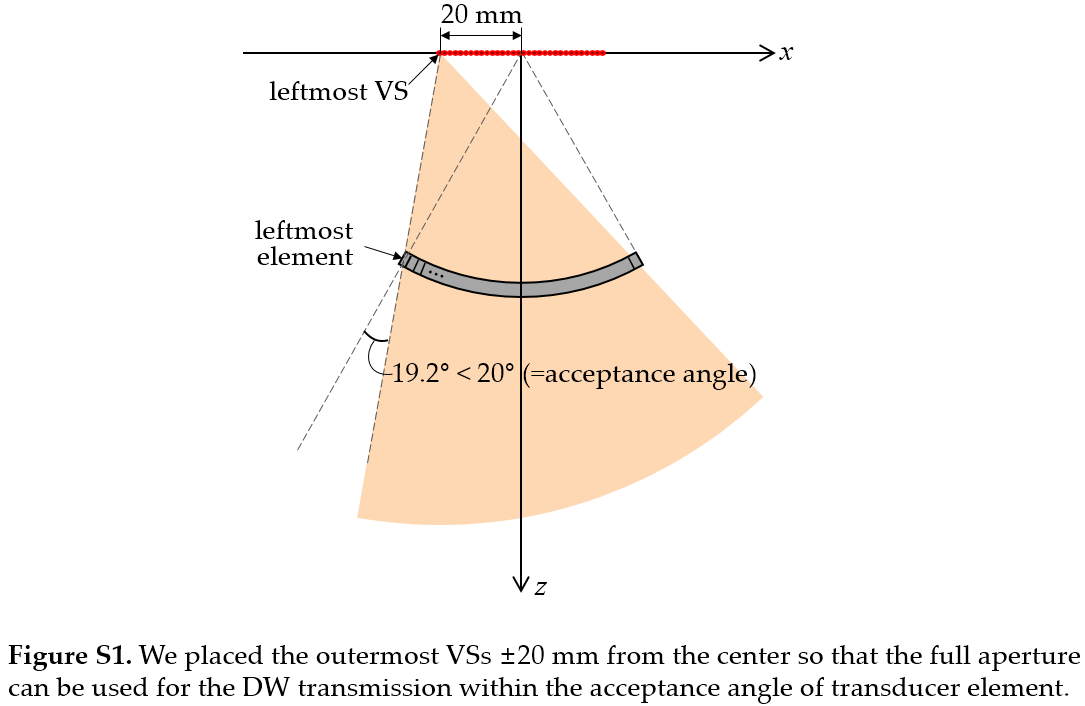

Supplement: Supplementary file 1 [file sensors-20-05675-s001.zip › Figure S1.png]
